# Association between blood pressure multi-trajectory and cardiovascular disease among a Chinese elderly medical examination population

**DOI:** 10.3389/fcvm.2024.1363266

**Published:** 2024-07-24

**Authors:** Quan Chen, Jinling Du, Xin Hong

**Affiliations:** ^1^ Department of Noncommunicable Disease Prevention, Nanjing Center for Disease Control and Prevention Affiliated to Nanjing Medical University, Nanjing, Jiangsu, China; ^2^ Department of Education, Wuxi No. 2 People's Hospital, Wuxi, Jiangsu, China; ^3^ Office of Operations Management, Guangzhou Liwan Center for Disease Control and Prevention, Guangzhou, Guangdong, China

**Keywords:** elderly population, group-based trajectory model, multivariate trajectory, blood pressure, cardiovascular disease

## Abstract

**Objective:**

This study aimed to characterize multivariate trajectories of blood pressure [systolic blood pressure (SBP) and diastolic blood pressure (DBP)] jointly and examine their impact on incident cardiovascular disease (CVD) among a Chinese elderly medical examination population.

**Methods:**

A total of 13,504 individuals without CVD during 2018–2020 were included from the Chinese geriatric physical examination cohort study. The group-based trajectory model was used to construct multi-trajectories of systolic blood pressure and diastolic blood pressure. The primary outcome was the incidence of the first CVD events, consisting of stroke and coronary heart diseases, in 2021. The Cox proportional hazards model was used to calculate the hazard ratios (HRs) and 95% confidence intervals (CIs) for the association between BP multi-trajectories and incident CVD events.

**Results:**

We identified four blood pressure (BP) subclasses, summarized by their SBP and DBP levels from low to high as class 1 (7.16%), class 2 (55.17%), class 3 (32.26%), and class 4 (5.41%). In 2021, we documented 890 incident CVD events. Compared with participants in class 1, adjusted HRs were 1.56 (95% CI: 1.12–2.19) for class 2, 1.75 (95% CI: 1.24–2.47) for class 3, and 1.88 (95% CI: 1.24–2.85) for class 4 after adjustment for demographics, health behaviors, and metabolic index. Individuals aged 65 years and above with higher levels of BP trajectories had higher risks of CVD events in China.

**Conclusions:**

Individuals with higher levels of both SBP and DBP trajectories over time were associated with an increased risk of incident CVD in the Chinese elderly population.

## Highlights

• Longitudinal data for systolic and diastolic blood pressure were modeled to identify four distinct multivariate trajectories of blood pressure function in older Chinese adults.
• Higher-level multi-trajectory groups had a significantly increased risk of cardiovascular disease.

## 1 Introduction

Cardiovascular disease (CVD) is the leading cause of death and disease burden in China, accounting for more than 40% of deaths (1), which means two out of every five deaths are due to CVD (2). The prevalence of CVD in China is continuously rising, with the number of patients now reaching 330 million (3). Data from the 2019 global burden of disease show that between 1990 and 2019, the number of CVD deaths among people aged 65 years and older in China increased from 1.577 to 3.404 million, increasing from 65.1% to 74.3% of total deaths (4, 5). In conclusion, CVD is a growing public health concern. Approaches focused on modifiable risk factors to prevent CVD are essential to control this serious situation.

High blood pressure (BP) is a well-recognized and widely accepted risk factor for the development of CVD (6–8). A report based on experience in 61 cohort studies showed (9) that the risk of CVD was two-fold higher for a 20 mmHg higher level of systolic blood pressure (SBP) and 10 mm Hg higher level of diastolic blood pressure (DBP). Previous cohort studies showed that an increasing trend in SBP had a higher risk of CVD (10, 11). Although hypertension (HTN) is a risk factor for a wide range of diseases, it is reversible, and adverse outcomes are preventable. For example, epidemiological evidence showed that every 10 mmHg reduction in SBP can reduce the risk of stroke by 41% (33%–48%) and coronary heart disease (CHD) by 22% (17%–27%) (12). One analysis of a large-scale epidemiological dataset demonstrated that a reduction in DBP is associated with a reduced risk for CVD events in individuals with isolated diastolic hypertension (IDH) (13). In general, both SBP and DBP play an important role in the development of CVD.

The longitudinal changes in BP in relation to CVD incidence have been gaining popularity recently (14–18). Xu et al. conducted a study only assessing the association between trajectories of SBP and CVD, leaving out the association between DBP and CVD (11). Obtaining the multivariate trajectory of SBP and DBP jointly by multiple measurements can more accurately predict the risk of CVD, which is useful for early identification and prevention of CVD incidence. Group-based trajectory modeling (GBTM) is a grouping method that can identify populations with similar processes of metabolic indicators over time (19). It has been well established that lifestyle (20) and health conditions (21) are associated with an increased risk of CVD outcomes; therefore, the confounding or covariate influences of these factors on the association between BP and incident CVD should be considered.

By using repeated measurements of SBP and DBP from the Chinese elderly checkup cohort during 2018–2020, our study aimed to jointly explore common latent classes and patterns of hypertension profiles based on multivariate trajectory analysis and examine the association of SBP and DBP multivariate trajectories with incident CVD. Therefore, this study may have important implications for improving disease prevention or treatment strategies.

## 2 Methods

### 2.1 Study design and populations

The data used in this study were derived from the “Nanjing Regional Health Information Platform based on Health Records.” Briefly, the study is a community-based and prospective cohort study in Nanjing, located in southern China. We randomly selected the Pukou District, covering all streets with each street covering all neighborhood/village committees. From 2018 to 2020, a total of 24,537 participants were recruited. They completed a questionnaire through face-to-face interviews, which included demographic variables, lifestyle factors, and routine medical examinations.

**Figure 1:**
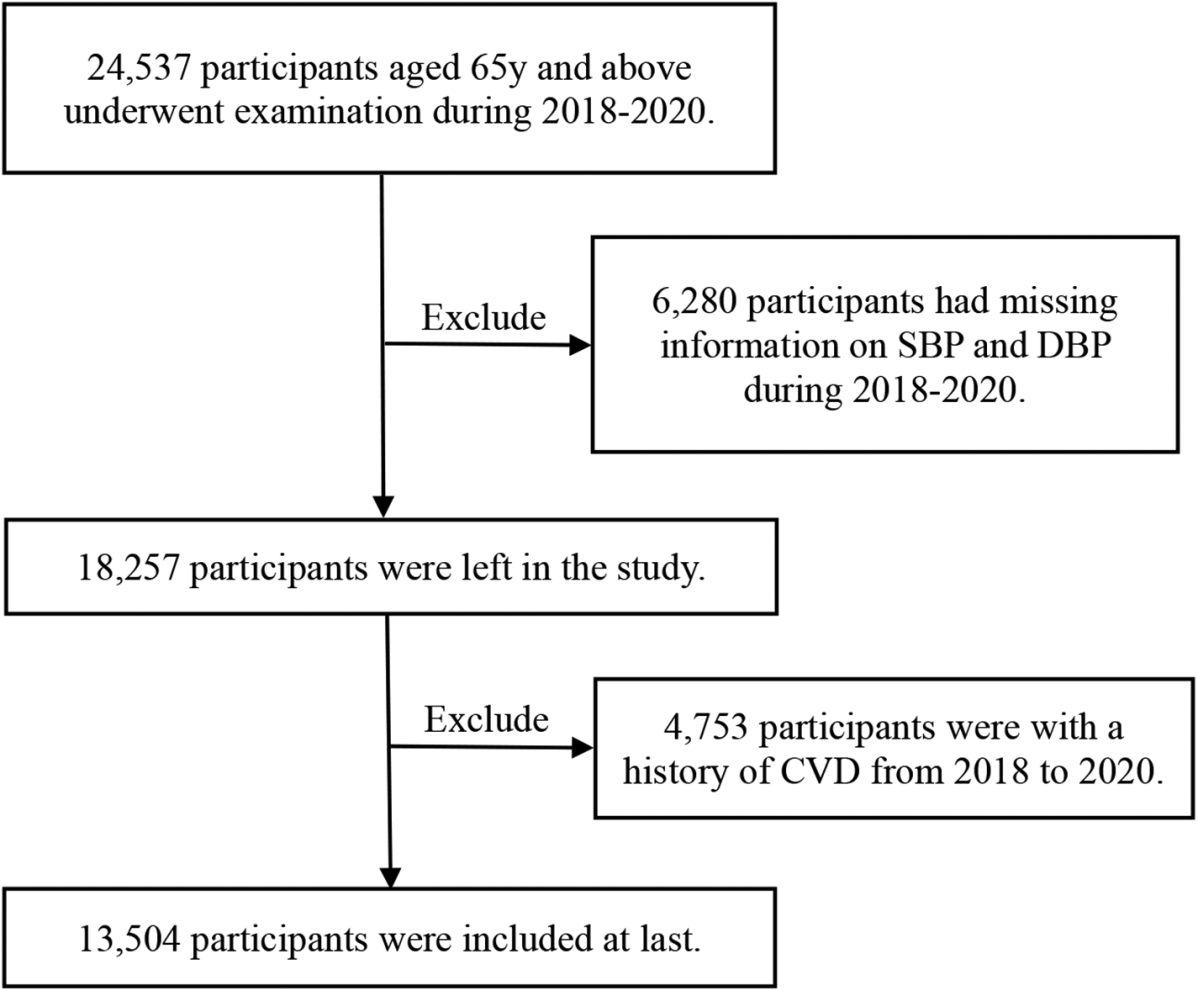
Flowchart of inclusion of participants.

Individuals who met all the following criteria were included: (1) aged 65 years and above at the first health checkup; (2) attended all health checkups between 2018 and 2020 with complete SBP and DBP data on three occasions; (3) had no diagnosis of CVD in any of health checkups from 2018 to 2020. The final sample size of this study was 13,504 (Figure 1). Baseline characteristics of the excluded and included populations were shown in Supplementary Table S1.

### 2.2 Data collection and measurement

Data were collected based on the National Basic Public Health Service Specification (3rd edition), including the personal basic information form and health examination form. The personal basic information form included their demographic characteristics (age, sex, marital status, and education). The health examination form included physical measurements, health behaviors (smoking, drinking), complementary examinations, and medical history. Medical history was obtained by trained research staff.

Physical measurements included height, body weight, waist circumference (WC), and BP. Waist circumference was measured to the nearest 0.1 cm at the midpoint between the lowest rib margin and the iliac crest following a standard protocol. Height was measured to the nearest 0.1 cm using a tape rule, and weight was measured to the nearest 0.1 kg using calibrated platform scales, both with the participant standing erect and barefoot. Body mass index (BMI) was calculated by dividing weight (kg) by height squared (m^2^). BP was measured three times by trained medical staff using the Omron HBP-1300 electronic BP monitors at each visit after a 10-min seated rest. We calculated the average of three measurements of SBP and DBP, respectively. The laboratory examination includes fasting plasma glucose (FPG), total cholesterol (TC), triglycerides (TGs), high-density lipoprotein cholesterol (HDL-C), and low-density lipoprotein cholesterol (LDL-C).

### 2.3 Main outcomes

The main outcome was the incidence of the first CVD events, consisting of stroke and CHD. We used the International Classification of Diseases, Tenth Revision (ICD-10) clinical codes to identify cases. Patients with ICD-10 codes from I00 to I99 were considered to have CVD. Stroke included hemorrhagic stroke (I60–I62.9, I67.0–I67.1, I68.1–I68.2, I69.0–I69.2) and ischemic stroke (G45–G46.8, I63–I63.9, I65–I66.9, I67.2–I67.3, I67.5–I67.6, I69.3). CHD included I20, I21, I22, I23, I24, and I25 (22), which denote angina pectoris, acute myocardial infarction, subsequent myocardial infarction, complications after myocardial infarction, other acute ischemic heart diseases, and chronic ischemic heart diseases, respectively. The diagnostic criteria were adopted from the 2018 edition of the diagnostic guidelines (23–25). Medical histories of CVD were collected in the database, and the diagnosis date of CVD was defined as the earliest record.

### 2.4 Other covariates and definitions

Age was categorized into two groups: 65–79 and ≥80 years. Marital status was categorized as follows: currently married and living with the spouse and divorced, widowed, or not married. Education was categorized as follows: less than lower secondary and lower secondary or above.

Drinking was defined as consuming more than 0.1 serving/day, and smoking was defined as smoking at least one cigarette a day on average in the past year. Based on self-reported smoking and drinking status, participants were classified as follows: non-smokers (including previous smokers) or current smokers; non-drinkers (including previous drinkers) or current drinkers. Obesity was defined as BMI ≥ 28 kg/m^2^ (26). Diabetes mellitus was defined as FPG ≥ 7.0 mmol/L, current treatment with a hypoglycemic agent, or self-reported physician diagnosis of diabetes mellitus (27). Hyperlipidemia was defined as the presence of any one of the following four situations: TG ≥ 2.26 mmol/L, TC ≥ 6.22 mmol/L, LDL-C ≥ 4.14 mmol/L, or HDL-C ≤ 1.04 mmol/L (28).

### 2.5 Establish the trajectory model

To categorize the trend of BP over time, we applied GBTM. The longitudinal SBP and DBP data were fitted as trajectories in a multivariate censored normal (CNORM) model (29). Multi-trajectories of SBP and DBP mean that each individual in a group has two trajectory curves: one for SBP and the other for DBP. Starting with a one-group model, we evaluated the optimal number of groups and polynomial type of each group trajectory (intercept, linear, or quadratic) with the following criteria: (1) Bayesian information criterion (BIC), where a value closer to 0 indicates a better fit model; (2) the value of the Bayesian factor logarithm, which is approximately two times the difference in BIC between the two compared models; (3) allocation of at least >5% of the total patients in each trajectory group in a model; and (4) higher average probability of final group membership across the trajectory groups.

As the number of trajectory groups was >4, although the Bayesian information criterion was closer to 0, the value of Bayesian factor logarithm was bigger (Supplementary Table S2), and the number of participants in one group was <5% of total. Finally, four distinct trajectories with regard to changes in both SBP and DBP were identified: 967 (7.16%), 7,450 (55.17%), 4,356 (32.26%), and 731 (5.14%) patients were assigned to the class 1, class 2, class 3, and class 4 trajectory groups, respectively. Supplementary Tables S2 and S3 present related parameters of the optimal multivariate trajectories of SBP and DBP after several attempts.

### 2.6 Statistical analyses

SPSS 26.0 and SAS 9.4 software were used for statistical analysis. Our statistical analysis mainly included two steps. Initially, multivariate trajectories of SBP and DBP from 2018 to 2020 were identified by using grouped trajectory modeling within the PROC TRAJ procedure in SAS (30) that share similar underlying BP patterns, which is an innovative statistical method to identify subgroups of participants who are homogeneous with the trajectory but heterogeneous compared with other trajectories.

Baseline characteristics of the participants are expressed as means and standard deviations (SDs) for continuous variables and as percentages for categorical variables. Characteristics across different groups were compared using analysis of variance or Kruskal–Wallis tests for continuous variables and *χ*^2^ statistics for categorical variables. Hazards ratios (HRs) and 95% confidence intervals (CIs) for incident CVD according to BP trajectories were analyzed by Cox proportional hazards regression models using class 1 as the reference. A two-sided *P* < 0.05 was regarded as significantly different.

Demographic characteristics, health behaviors, and biological data were addressed as control variables. Four models were estimated: model 1, an unadjusted model; model 2, adjusted for demographic characteristics; model 3, further adjusted for health behaviors; and model 4, further adjusted for biological data based on model 3.

### 2.7 Ethical approval

This current study was approved by the Ethics Committee of Nanjing Center for Disease Control and Prevention (PJ2022002), and all participants voluntarily participated in this study and provided written informed consent.

## 3 Results

### 3.1 Basic information about the research object

Table 1 presents the baseline characteristics of 13,504 study participants. At baseline, the mean age was 71.94 (SD = 5.85) years, and 55.79% of the participants were women. Over half of the study participants were divorced, widowed, or not married (69.64%). There were 17.61% current smokers and 17.77% current drinkers. The mean and SD values of metabolic indicators and baseline prevalence of dyslipidemia, diabetes, and obesity are also listed in Table 1. During the observation period, the incidence of CVD was 6.59% (890/13,504).

**Table 1:** Baseline characteristics of the study participants (*N* = 13,504).

Characteristics	*N* (%) or mean ± SD
Age (mean ± SD, years)	71.94 ± 5.85
Age (years), *n* (%)	
65–79	11,798 (87.37)
≥80	1,706 (12.63)
Sex, *n* (%)	
Male	5,970 (44.21)
Female	7,534 (55.79)
Marital status, *n* (%)	
Married	4,100 (30.36)
Divorced, widowed, or not married	9,404 (69.64)
Education, *n* (%)	
Lower secondary or above	2,275 (16.85)
Less than lower secondary below	11,229 (83.15)
Smoker, *n* (%)	
Never or previous	11,126 (82.39)
Current	2,378 (17.61)
Drinker, *n* (%)	
Never or previous	11,104 (82.23)
Current	2,400 (17.77)
BMI (mean ± SD, kg/m^2^)	24.59 ± 3.38
WC (mean ± SD, cm)	84.10 ± 9.37
SBP (mean ± SD, mmHg)	142.36 ± 18.45
DBP (mean ± SD, mmHg)	81.78 ± 10.01
FPG (mean ± SD, mmol/L)	5.65 ± 1.64
TC (mean ± SD, mmol/L)	4.83 ± 0.97
TG (mean ± SD, mmol/L)	1.71 ± 1.33
LDL-C (mean ± SD, mmol/L)	2.78 ± 0.81
HDL-C (mean ± SD, mmol/L)	1.64 ± 0.50
Obesity, *n* (%)	2,058 (15.24)
Diabetes mellitus, *n* (%)	2,524 (18.69)
Dyslipidemia, *n* (%)	2,634(19.51)

### 3.2 Track grouping results

**Figure 2:**
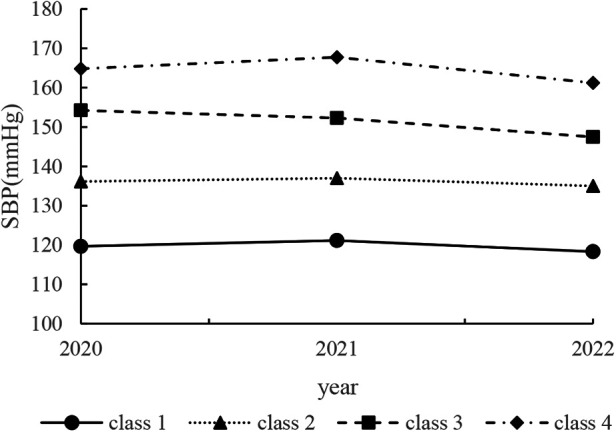
Predicted trajectories of four systolic blood pressure value classes among elderly Chinese adults, Nanjing Regional Health Information Platform Based on Health records 2018–2020 (*N* = 13,504). The *Y*-axis indicates the systolic blood pressure value. The *X*-axis indicates the year.

**Figure 3:**
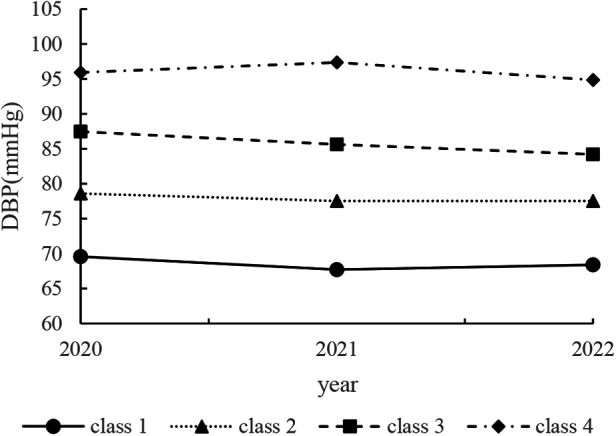
Predicted trajectories of four diastolic blood pressure value classes among elderly Chinese adults, Nanjing Regional Health Information Platform Based on Health Records 2018–2020 (*N* = 13,504). The *Y*-axis indicates the diastolic blood pressure value. The *X*-axis indicates the year.

Using the procedure and criteria mentioned above, we chose the four-group multi-trajectory model from all investigated models after several attempts. Figure 2 shows the plot of four distinct SBP trajectories by using the SAS PROC TRAJ program, and Figure 3 shows DBP trajectories. The four groups of trajectories were marked as class 1 (967, 7.16%), class 2 (7,450, 55.17%), class 3 (4,356, 32.26%), and class 4 (731, 5.41%). Related parameters of the four groups of trajectories were provided in Supplementary Tables S3 and S4.

As shown in Figures 2, 3, class 2 consisted of the highest number of individuals with moderate SBP and DBP (mean SBP ranged from 135.02 to 137.01 mmHg and mean DBP ranged from 77.54 to 78.62 mmHg in 2018–2020). By contrast, class 4 had the fewest participants with the highest SBP and DBP (mean SBP ranged from 161.26 to 167.78 mmHg and mean DBP ranged from 94.86 to 97.38 mmHg in 2018–2020) at the same time. Participants in class 1 initially showed an elevated SBP that decreased over time (mean SBP ranged from 118.32 to 121.14 mmHg in 2018–2020), while their DBP decreased and then increased (mean DBP ranged from 94.86 to 97.38 mmHg in 2018–2020). Participants in class 3 consistently maintained lower indexes of SBP and DBP (mean SBP decreased from 154.26 to 147.47 mmHg and mean DBP decreased from 87.48 to 84.21 mmHg in 2018–2020).

### 3.3 Characteristics of different trajectory groups

Demographic data of the four BP trajectory groups are provided in Table 2. Age, gender, level of education, and biological data, including BMI, WC, SBP, DBP, FPG, TG, LDL-C, and HDL-C, differed notably among the trajectory groups (all *P*’s* *< 0.05). Participants in the higher BP trajectory groups were more likely to be current drinkers at baseline (*P *< 0.05). In terms of metabolic metrics, participants in the higher BP trajectory groups had higher levels of BMI, WC, FPG, and LDL-C than those in lower BP trajectory groups (all *P*’s* *< 0.05). Participants in the highest BP trajectory group were more likely to be obese and have dyslipidemia (all *P*’s* *< 0.05).

**Table 2:** Characteristics of the study population by blood pressure classes.

Variables	Blood pressure classes				*F*/*χ*^2^	*P*-value *
	Class 1	Class 2	Class 3	Class 4		
*N* (%)	967 (7.16)	7,450 (55.17)	4,356 (32.26)	731 (5.41)		
Age (mean ± SD, years)	71.17 ± 5.40	72.22 ± 6.10	71.80 ± 5.61	71.01 ± 4.95	18.31	<0.01
Gender, *n* (%)					19.41	<0.01
Male	387 (40.02)	3,249 (43.61)	1,969 (45.20)	365 (49.93)		
Female	580 (59.98)	4,201 (56.39)	2,387 (54.80)	366 (50.07)		
Marital status, *n* (%)					0.37	0.95
Married	299 (30.92)	2,247 (30.16)	1,331 (30.56)	223 (30.51)		
Divorced, widowed, or not married	668 (69.08)	5,203 (69.84)	3,025 (69.44)	508 (69.49)		
Education status, *n* (%)					15.72	<0.01
Lower secondary or above	145 (14.99)	1,332 (17.88)	699 (16.05)	99 (13.54)		
Less than lower secondary below	822 (85.01)	6,118 (82.12)	3,657 (83.95)	632 (86.46)		
Smoker, *n* (%)					8.09	0.04
Never or previous	772 (79.83)	6,174 (82.87)	3,594 (82.51)	586 (80.16)		
Current	195 (20.17)	1,276 (17.13)	762 (17.49)	145 (19.84)		
Drinker, *n* (%)					112.68	<0.01
Never or previous	842 (87.07)	6,299 (84.55)	3,414 (78.37)	549 (75.10)		
Current	125 (12.93)	1,151 (15.45)	942 (21.63)	182 (24.90)		
BMI (mean ± SD, kg/m^2^)	23.00 ± 3.31	24.40 ± 3.27	25.15 ± 3.40	25.34 ± 3.58	134.05	<0.01
WC (mean ± SD, cm)	81.09 ± 9.70	83.78 ± 9.16	85.13 ± 9.39	85.28 ± 9.72	58.28	<0.01
SBP (mean ± SD, mmHg)	119.65 ± 13.59	136.16 ± 13.51	154.26 ± 15.54	164.83 ± 17.60	2,832.51	<0.01
DBP (mean ± SD, mmHg)	69.57 ± 7.23	78.62 ± 7.50	87.48 ± 8.36	95.94 ± 9.50	2,717.01	<0.01
FPG (mean ± SD, mmol/L)	5.44 ± 1.74	5.62 ± 1.59	5.72 ± 1.59	5.81 ± 1.72	11.30	<0.01
TC (mean ± SD, mmol/L)	4.77 ± 1.38	4.93 ± 5.04	4.94 ± 1.51	4.97 ± 0.90	0.56	0.64
TG (mean ± SD, mmol/L)	1.29 ± 0.97	1.44 ± 1.64	1.55 ± 1.25	1.53 ± 1.01	10.36	<0.01
LDL-C (mean ± SD, mmol/L)	2.66 ± 0.80	2.76 ± 0.75	2.84 ± 0.79	2.91 ± 0.85	25.03	<0.01
HDL-C (mean ± SD, mmol/L)	1.69 ± 0.71	1.67 ± 0.78	1.63 ± 0.50	1.66 ± 0.73	3.72	0.01
Obesity, *n* (%)	64 (6.62)	1,017 (13.65)	822 (18.87)	155 (21.20)	134.78	<0.01
Diabetes mellitus, *n* (%)	157 (16.24)	1,479 (19.85)	763 (17.52)	125 (17.10)	15.62	<0.01
Dyslipidemia, *n* (%)	151 (15.62)	1,279 (17.17)	1,020 (23.42)	184 (25.17)	92.62	<0.01
CVD incidence, *n* (%)	38 (3.92)	467 (6.27)	327 (7.51)	58 (7.93)	20.46	<0.01

* *P-*value was based on the chi-square test, ANOVA, or the Kruskal–Wallis test where appropriate.

In 2021, 890 cases (6.59%) of cardiovascular disease were newly reported. Among them, there were 38, 467, 327, and 58 cases in class 1, class 2, class 3, and class 4, respectively, accounting for 3.92%, 6.27%, 7.51%, and 7.93% of the total number of participants in each group. Also, the difference was statistically significant (all *P*’s* *< 0.05). Supplementary Table S5 presents the baseline characteristics of the population with or without CVD.

### 3.4 Association of different trajectory groups and cardiovascular diseases

Table 3 presents the risks of developing CVD under each influencing factor by using a one-factor Cox proportional risk regression model. Many previous studies (11, 20) have shown that demographic characteristics (age, sex, marital status, and education), health behaviors (smoking, drinking), and biological data (BMI, WC, FPG, TC, TG, LDL-C, and HDL-C) are all contributors to CVD. In our study, sex, education, drinking status, BMI, WC, and FPG were risk factors for CVD (all HRs* *> 1, *P *< 0.05). HDL-C was found to be a protective factor against CVD development (HR* *< 1, *P *< 0.05).

**Table 3:** One-way Cox proportional risk regression model of the risk of developing CVD.

Variables	*B*	*SE*	Wald *χ^2^*	*P*	HR (95% CI)	* *
Sex	0.35	0.07	25.00	<0.01	1.42 (1.24–1.63)	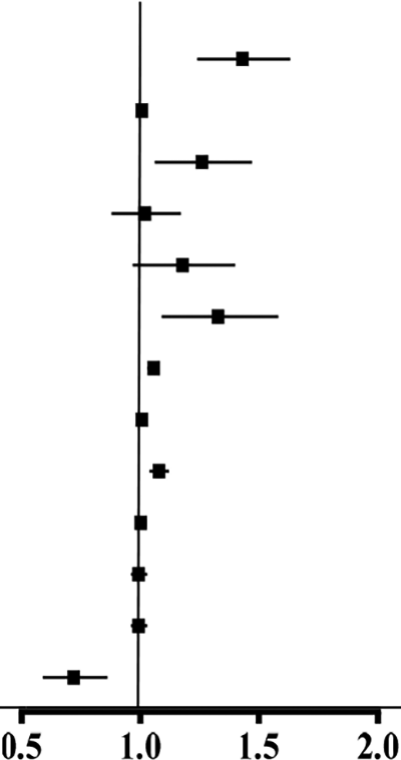
Age	0.01	0.01	0.67	0.41	1.01 (0.99–1.02)	
Education	0.22	0.08	7.221.4	<0.01	1.25 (1.06–1.47)	
Marital status	0.01	0.07	0.03	0.87	1.01 (0.88–1.17)	
Smoking	0.15	0.09	2.71	0.10	1.17 (0.97–1.40)	
Drinking	0.27	0.10	7.91	<0.01	1.31 (1.09–1.58)	
BMI	0.05	0.01	30.84	<0.01	1.06 (1.03–1.08)	
WC	0.01	0.00	10.30	<0.01	1.01 (1.00–1.01)	
FPG	−0.01	0.01	14.21	<0.01	1.08 (1.04–1.12)	
TG	0.00	0.01	0.01	0.94	1.00 (0.99–1.02)	
TC	−0.01	0.02	0.09	0.76	0.99 (0.96–1.03)	
LDL-C	−0.01	0.02	0.14	0.71	0.99 (0.96–1.03)	
HDL-C	−0.34	0.10	12.97	<0.01	0.71 (0.59–0.86)	

**Figure 4:**
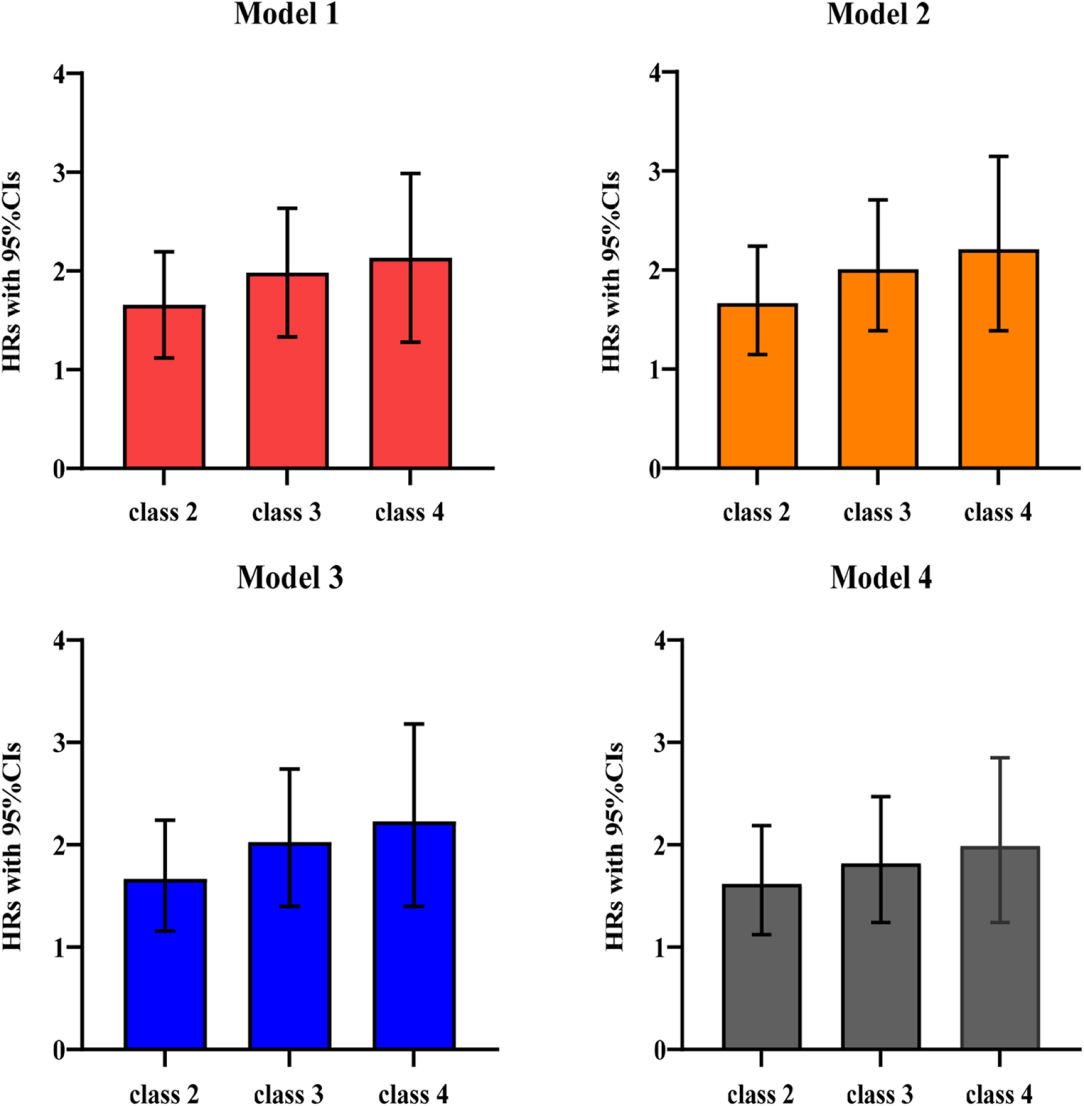
HRs and 95% CIs for CVD by multivariate trajectories of blood pressure in older adults in China, 2018–2020 (*N* = 13,504). Estimates are weighted using the sampling weight. Model 1: unadjusted. Model 2: adjusted for baseline sex and education. Model 3: model 2 further adjusted for drinking status. Model 4: model 3 further adjusted for BMI, WC, FPG, and HDL-C.

Multi-trajectories of BP were significantly associated with CVD during the follow-up period across four models. Figure 4 shows the HRs and 95% CIs for CVD by multivariate trajectories of BP. Compared with class 1, the CVD outcome events in class 2, class 3, and class 4 increased by 60% (95% CI: 1.15–2.22), 91% (95% CI: 1.37–2.67), and 102% (95% CI: 1.34–3.04), respectively, in 2021 (Supplementary Table S6). After adjusting for gender and education (model 2), the risk of CVD in class 2, class 3, and class 4 increased by 61% (95% CI: 1.15–2.24), 94% (95% CI: 1.39–2.71), and 109% (95% CI: 1.39–3.15), respectively, compared with that in class 1 (Supplementary Table S7). In model 3, which was further adjusted for drinking status in model 2, compared with class 1, class 2 (HR* *= 1.61, 95% CI: 1.16–2.24), class 3 (HR* *= 1.95, 95% CI: 1.40–2.74), and class 4 (HR* *= 2.11, 95% CI: 1.40–3.18) were significantly associated with an increased risk of CVD during the follow-up period in older Chinese adults (Supplementary Table S8). The HRs of class 2, class 3, and class 4 for the occurrence of CVD were 1.56 (95% CI: 1.12–2.19), 1.75 (95% CI: 1.24–2.47), and 1.88 (95% CI: 1.24–2.85) after further adjusting for BMI, WC, FPG, and HDL-C in model 4 (based on model 3). Compared with class 1, the risk of cardiovascular disease in class 2, class 3, and class 4 gradually increased with the elevation of SBP and DBP levels, and the risk of CVD in model 2, model 3, and model 4 still had a similar trend among different trajectory groups after adjusting for confounding factors (Supplementary Table S9).

## 4 Discussion

In this cohort study of 13,504 participants, we investigated heterogeneity in multivariate BP trajectories among individuals aged 75.94 ± 5.85 years in China. Using group-based multi-trajectory analyses of the longitudinal data across the three examination cycles, we identified four distinct multivariate trajectories of SBP and DBP levels. The risk of CVD increased with the levels of SBP and DBP trajectories. Compared to the low-stable class (class 1), individuals with higher levels of BP had a 1.60–2.02-fold risk of CVD.

Previously, a large body of epidemiological evidence has shown that BP is associated with cardiovascular morbidity (31). Fan and colleagues studied the association between BP and mortality risk in a Chinese county by using measurements of BP at two time points (at the beginning and end of observation) (32). They found that relative to stable BP of normotension, an increase in BP from normotension to hypertension or from prehypertension to hypertension increased the risk of CVD. By contrast, our study used GBTM to identify multi-trajectories of BP and corroborated previous findings. This approach was superior to the method applied by Fan et al. BP information for every year was comprehensively considered in our study instead of only two measurements used in the study conducted by Fan et al. Tielemans et al. (33) identified four SBP trajectories, in which mean SBP increased by 5–12 mmHg for 10 years. The highest trajectories were associated with two to three times greater CVD mortality and a 1.5 times higher risk of all-cause mortality compared with the lowest trajectory. Although DBP has also been recognized as an important independent risk factor for CVD (34), there has been relatively less research on metrics that combine the information about SBP and DBP to simultaneously examine their effects. Our study suggests that assessing both SBP and DBP may be a better predictor of CVD risk factors compared with assessing SBP or DBP alone.

According to the Chinese guidelines for the prevention and treatment of hypertension in adults (2018 Revised Edition) (35), BP is classified as follows: normal (SBP < 120 mmHg and DBP < 80 mmHg), high normal (SBP ranged 120–139 mmHg and/or DBP ranged 80–89 mmHg), and hypertension (SBP ≥ 140 mmHg and/or DBP ≥ 90 mmHg). We found that the mean SBP of the participants in the lowest trajectory groups was 121.14 mmHg during the second health checkup, which categorizes them as having high normal BP. Class 2, consisting of the majority of participants (55.17%), all had high normal SBP values; participants of class 3 and class 4 were all classified as hypertensive according to these guidelines. General knowledge dictates that BP increases with age. This elevation in BP among the elderly may be a compensatory mechanism to guarantee normal blood circulation to the heart, brain, and kidneys, which may explain the higher BP levels among participants aged 65 years and above in our study. HTN is one of the main contributive factors to CVD (36). A meta-analysis declared that prehypertension (SBP ranged 120–140 mmHg and/or DBP ranged 80–90 mmHg) is significantly associated with a higher risk of CVD events (37). A Chinese study reported that compared to those who maintained BP <130/80 mmHg, those remaining in stage 1 (SBP 130–139 mmHg or DBP 80–89 mmHg) and progressing to stage 2 HTN (SBP ≥ 140 mmHg or DBP ≥ 90 mmHg) had a higher risk of CVD (38). As the mean SBP and DBP of multi-trajectories increased in class 2, class 3, and class 4 in our study, the participants exhibited a higher risk of CVD compared with those in class 1. The findings from our study appeared relatively consistent with previous studies on this topic.

It should be noticed that participants in class 3 consistently maintained decreased SBP and DBP. They might have implemented standardized and effective hypertension control programs to achieve a sustained reduction in BP over the 3-year period. Participants in class 4 initially had elevated SBP and DBP, which then decreased. A possible explanation for this might be that as BP levels worsen, individuals become more concerned about their health and take action to control BP. Our research showed that class 3 and class 4 still had a higher risk of CVD, possibly influenced by sustained higher levels of BP. Even though participants in class 2 had moderately stable SBP and DBP, they still had a higher risk of CVD than those in the low-fluctuant class (class 1). It is, therefore, important to identify individuals with moderate stable trajectories but normal high SBP levels early on, allowing for instituting targeted preventive measures to reduce the risk of CVD.

With a rapidly aging global population and changing disease epidemiology, CVD remains a significant cause of both morbidity and mortality globally, especially among older adults (39, 40). We found that the incidence of CVD in this Chinese elderly cohort was 6.59% (890/13,504) during the follow-up period, much higher than 867.65/100,000 in 2019 (41). Bress et al. reported that 60% and 33% of the development of high 10-year predicted CVD risk is attributable to aging and increased SBP, respectively (42). Increasing age increases CVD risk directly and indirectly by worsening risk factors such as lipids and BP (31, 43, 44). Our study focused on Chinese elderly medical checkups, highlighting that structural and functional changes in the cardiovascular system compromise cardiac reserve, predisposing individuals to CVD (45).

The occurrence and development of CVD result from a combination of multiple risk factors, including age, gender, overweight and obesity, hypertension, dyslipidemia, and hypoglycemia (7, 46). Therefore, the present study, even after adjusting for cardiovascular health metrics (i.e., sex, education, drinking, BMI, WC, FPG, and HDL-C), still concluded that the risk of CVD development was higher in the trajectory groups with slight higher levels of SBP and DBP values than in the groups with lower levels. Our study found that the association between the BP multi-trajectory group and incident CVD events was not moderated by sex, education, drinking status, BMI, WC, FPG, and HDL-C. Meanwhile, the trajectories that we identified extend results from prior studies of BP trajectories in young- to middle-aged people. Those studies identified three to six parallel trajectories in which trajectories with long-term higher BP related to more cardiovascular pathology (47–49). In our older population, we also observed that the class with higher BP levels had a higher risk of stroke and death compared with the class with the lowest BP trajectory. Portegies et al. fitted four SBP trajectories across ages ranging from 55 to 106 years (17). They found that high but decreasing BP and rapidly increasing BP patterns were associated with a high risk of stroke and death, whereas moderately high BP is only related to an increased risk of stroke. Li et al. found that a trajectory of moderately rising and then falling or high levels of falling BP was a potentially critical period for the development of CHD (50). Our findings were in accord with those studies indicating that moderate BP, moderately high but decreasing BP, and high-fluctuant BP curves had significantly higher risks of CVD compared with the lowest BP trajectory class. Norby et al. reported four hypertension trajectories, while Zheng et al. identified four trajectories each for SBP and DBP (51, 52). The number of trajectories they fitted was consistent with our study. However, they did not identify decreasing curves. A downward trajectory indicates that interventions to combat hypertension have been effective. Our findings of multi-trajectories of BP and the association between patterns of BP and risk of CVD could bring a clue for the cause, preventive policy, and treatment guidelines for the Chinese elderly population in the future.

The strengths of our study include its prospective design, repeated measures of BP, and a wide range of covariates that include demographics, health behaviors, and metabolic biomarkers. A key strength of this study lies in the use of an innovative multi-trajectory modeling technique to identify subgroups of longitudinal BP profile trajectories, which can effectively overcome the limitations of single measurement data analysis by establishing a sequence of the dynamic changes of an observed indicator over time and following a homogeneous developmental trajectory for grouping (53, 54). GBTM is a more common method of trajectory grouping, which can explore the number of subgroups included in the development of indicators of a population over time and their trends (55). Repeated BP measurements over a long exposure period can minimize the likelihood of reverse causation or the potential bidirectionality of the estimated association between BP and CVD events. However, this study has several limitations. First, the identification of CVD cases was based on medical records from the available database, which may be subject to reporting bias. Second, the observation time is too short to adequately reflect the long-term morbidity of the population, which may affect the stability of the results. Third, the study population was from a district in Nanjing, China, and the findings from our study might not be generalizable to the entire elderly population in China. Finally, we excluded individuals missing SBP/DBP values may bias the result. However, some evidence clarifies that at least three unique points are required for the trajectory approach (56, 57).

## 5 Conclusion

In summary, we have identified four distinct mufti-trajectories of BP and demonstrated elevated SBP and DBP were associated with an increased risk of incident CVD in the elderly Chinese population. BP management is important among the elderly population to prevent CVD in their later lives. The observations from this study emphasize the importance of strong public health efforts to control BP for CVD prevention.

## Data Availability

The original contributions presented in the study are included in the article/Supplementary Material, further inquiries can be directed to the corresponding author.
