# Supplementary material for: Association between blood pressure multi-trajectory and cardiovascular disease among a Chinese elderly medical examination population
Source: Front Cardiovasc Med. 2024 Jul 24;11:1363266. doi: 10.3389/fcvm.2024.1363266 (PMC11303174; doi:10.3389/fcvm.2024.1363266)
Supplement: Supplementary file 1 [file Datasheet1.pdf]

Supplementary Files

Table S1. Baseline characteristics of excluded and included population (N=24,357)

Variables	Excluded(n=11033)	Included(n=13504)	F/χ^2	*P* value
Age (years), n (%)			1.65	0.20
65-79	9699(87.91)	11798(87.37)		
≥80	1334(12.09)	1706(12.63)		
Sex, n (%)			3.54	0.06
Male	5010(45.41)	5970(44.21)		
Female	6023(54.59)	7534(55.79)		
Marital status, n (%)			2.51	0.11
Married	3247(29.43)	4100(30.36)		
Divorced, widowed, or not married	7786(70.57)	9404(69.64)		
Education, n (%)			3.61	0.06
Lower secondary or above	1759(15.94)	2275(16.85)		
Less than lower secondary below	9274(84.06)	11229(83.15)		
Smoker, n (%)			0.23	0.63
Never or previous	9116(82.62)	11126(82.39)		
Current	1917(17.38)	2378(17.61)		
Drinker, n (%)			0.55	0.46
Never or previous	9032(81.86)	11104(82.23)		
Current	2001(18.14)	2400(17.77)		
BMI (mean±SD, kg/m^2)	25.02±3.32	24.59±3.38	97.61	<0.01
WC (mean±SD, cm)	84.33±9.43	84.10±9.37	3.53	0.06
SBP (mean±SD, mmHg)	142.66±18.00	142.36±18.45	1.44	0.23
DBP (mean±SD, mmHg)	81.73±10.11	81.78±10.01	0.15	0.70
FPG (mean±SD, mmol/l)	5.79±1.66	5.65±1.64	40.80	<0.01
TC (mean±SD, mmol/l)	4.88±0.99	4.83±0.97	102.64	<0.01
TG (mean±SD, mmol/l)	1.55±1.09	1.71±1.33	15.74	<0.01
LDL-C (mean±SD, mmol/l)	2.78±0.84	2.78±0.81	0.01	0.93
HDL-C (mean±SD, mmol/l)	1.62±0.44	1.64±0.50	9.11	<001

Table S2. Summary of Model Screening using a Group-based Trajectory Model of Blood Pressure among Older Adults in China, 2018-2020 (N=13,504)

Model/No. of Trajectory Groups	BIC	$2^*\Delta(BIC_{ij})$
1	-324054.4	(Baseline)
2	-318779.0	10550.8
3	-317544.1	2469.8
4	-317176.6	735.0
5	-316467.9	1417.4

Note: The 3-group model turn out to be the optimal, with lower BIC (-317176.6) and smaller incremental change ($2^*\Delta$ (BIC_{ij})= 735.0) at the same time. BIC=Bayesian Information Criterion.

Table S3. Related parameters of the four-group Trajectory Model of SBP among Older Adults in China, 2018-2020 (N=13,504)

SBP Group	Parameter	Estimate	Standard Error	T value	*P*
Class 1	Intercept	117.359	2.298	51.075	<0.001
	Linear	6.347	2.511	2.528	0.012
	Quadratic	-1.748	0.620	-2.818	0.005
Class 2	Intercept	132.185	0.932	141.881	<0.001
	Linear	6.233	0.966	6.453	<0.001
	Quadratic	-1.731	0.238	-7.281	<0.001
Class 3	Intercept	156.324	0.754	207.378	<0.001
	Linear	-3.157	0.260	-12.121	<0.001
Class 4	Intercept	147.348	3.043	48.422	<0.001
	Linear	20.830	3.405	6.118	<0.001
	Quadratic	-5.618	0.839	-6.698	<0.001

Table S4. Related parameters of the four-group Trajectory Model of DBP among Older Adults in China, 2018-2020 (N=13,504)

DBP Group	Parameter	Estimate	Standard Error	T value	*P*
Class 1	Intercept	74.584	1.261	59.124	<0.001
	Linear	-4.835	1.409	-3.431	<0.001
	Quadratic	1.064	0.349	3.051	0.002
Class 2	Intercept	81.316	0.484	167.901	<0.001
	Linear	-3.038	0.531	-5.720	<0.001
	Quadratic	0.620	0.131	4.729	<0.001
Class 3	Intercept	88.227	0.411	214.550	<0.001
	Linear	-1.561	0.138	-11.310	<0.001
Class 4	Intercept	88.304	1.726	51.167	<0.001
	Linear	8.656	1.933	4.477	<0.001
	Quadratic	-2.322	0.470	-4.242	<0.001

Table S5. Baseline characteristics of population with or without CVD (N=13,504)

Variables	Incident CVD Events		χ^2	*P* value
	Yes (n=890)	No (n=12614)		
Age (years), n (%)			11.47	<0.01
65-79	810(91.01)	10988(87.11)		
≥80	80(8.99)	1626(12.89)		
Sex, n (%)			27.04	<0.01
Male	319(35.84)	5651(44.80)		
Female	571(64.16)	6963(55.20)		
Marital status, n (%)			0.03	0.87
Married	268(30.11)	3832(30.38)		
Divorced, widowed, or not married	622(69.89)	8782(69.62)		
Education, n (%)			7.76	<0.01
Lower secondary or above	180(20.22)	2095(16.61)		
Less than lower secondary below	710(79.78)	10519(83.39)		
Smoker, n (%)			2.91	0.09
Never or previous	752(84.49)	10374(82.24)		
Current	138(15.51)	2240(17.76)		
Drinker, n (%)			8.52	<0.01
Never or previous	764(85.84)	10340(81.97)		
Current	126(14.16)	2274(18.03)		
Obesity, n (%)	189(21.24)	1869(14.82)	26.52	<0.01
Diabetes mellitus, n (%)	216(24.27)	2308(18.30)	19.51	<0.01
Dyslipidemia, n (%)	210(23.60)	2424(19.22)	10.15	<0.01

Table S6. HRs and 95% CI of blood pressure Multi-trajectory Classes on Incident CVD (Model 1)

BP trajectories	*B*	*SE*	*Wald*χ^2 *Value*	*P*	*HR (95%CI)*
Class 1			18.75		Reference
Class 2	0.47	0.17	7.66	<0.01	1.60(1.15-2.22)
Class 3	0.65	0.17	14.26	<0.01	1.91(1.37-2.67)
Class 4	0.70	0.21	11.33	<0.01	2.02(1.34-3.04)

Model 1: Unadjusted model.

Table S7. HRs and 95% CI of blood pressure Multi-trajectory Classes on Incident CVD (Model 2)

BP trajectories	*B*	*SE*	*Wald*χ^2 *Value*	*P*	*HR (95%CI)*
Class 1			20.30		Reference
Class 2	0.47	0.17	7.88	<0.01	1.61(1.15-2.24)
Class 3	0.66	0.17	14.93	<0.01	1.94(1.39-2.71)
Class 4	0.74	0.21	12.51	<0.01	2.09(1.39-3.15)

Model 2: Adjusting for baseline gender and education background.

Table S8. HRs and 95% CI of blood pressure Multi-trajectory Classes on Incident CVD (Model 3)

BP trajectories	*B*	*SE*	*Wald*χ^2 *Value*	*P*	*HR (95%CI)*
Class 1			20.93		Reference
Class 2	0.48	0.17	7.93	<0.01	1.61(1.16-2.24)
Class 3	0.67	0.17	15.25	<0.01	1.95(1.40-2.74)
Class 4	0.75	0.21	12.80	<0.01	2.11(1.40-3.18)

Model 3: Adjusted for variables in model 2 plus baseline drinking status (no or yes).

Table S9. HRs and 95% CI of blood pressure Multi-trajectory Classes on Incident CVD (Model 4)

BP trajectories	*B*	*SE*	*Wald*χ^2 *Value*	*P*	*HR (95%CI)*
Class 1			12.10		Reference
Class 2	0.45	0.17	6.74	<0.01	1.56(1.12-2.19)
Class 3	0.56	0.18	10.22	<0.01	1.75(1.24-2.47)
Class 4	0.63	0.21	8.84	<0.01	1.88(1.24-2.85)

Model 4: Adjusted for variables in model 3 plus BMI, WC, FPG and HDL-C.
